# Effect of Temperatures and Graphene on the Mechanical Properties of the Aluminum Matrix: A Molecular Dynamics Study

**DOI:** 10.3390/ma16072722

**Published:** 2023-03-29

**Authors:** Jingtao Huang, Mingwei Li, Jiaying Chen, Yuan Cheng, Zhonghong Lai, Jin Hu, Fei Zhou, Nan Qu, Yong Liu, Jingchuan Zhu

**Affiliations:** 1 School of Materials Science and Engineering, Harbin Institute of Technology, Harbin 150001, China; 2 National Key Laboratory for Precision Hot Processing of Metals, Harbin Institute of Technology, Harbin 150001, China; 3 National Key Laboratory of Science and Technology on Advanced Composites in Special Environments, Harbin Institute of Technology, Harbin 150001, China; 4 Center for Analysis, Measurement and Computing, Harbin Institute of Technology, Harbin 150001, China; 5 State Key Laboratory for Environment-Friendly Energy Materials, School of Materials Science and Engineering, Southwest University of Science and Technology, Mianyang 621010, China

**Keywords:** graphene, aluminium matrix composite, mechanical properties, tensile properties, molecular dynamics

## Abstract

Graphene has become an ideal reinforcement for reinforced metal matrix composites due to its excellent mechanical properties. However, the theory of graphene reinforcement in graphene/aluminum matrix composites is not yet well developed. In this paper, the effect of different temperatures on the mechanical properties of the metal matrix is investigated using a classical molecular dynamics approach, and the effects of the configuration and distribution of graphene in the metal matrix on the mechanical properties of the composites are also described in detail. It is shown that in the case of a monolayer graphene-reinforced aluminum matrix, the simulated stretching process does not break the graphene as the strain increases, but rather, the graphene and the aluminum matrix have a shearing behavior, and thus, the graphene “pulls out" from the aluminum matrix. In the parallel stretching direction, the tensile stress tends to increase with the increase of the graphene area ratio. In the vertical stretching direction, the tensile stress tends to decrease as the percentage of graphene area increases. In the parallel stretching direction, the tensile stress of the system tends to decrease as the angle between graphene and the stretching direction increases. It is important to investigate the effect of a different graphene distribution in the aluminum matrix on the mechanical properties of the composites for the design of high-strength graphene/metal matrix composites.

## 1. Introduction

As a two-dimensional carbon nanomaterial, graphene has extremely excellent mechanical, optical, and electro-catalytic properties [1,2] and high thermal conductivity [3] and is considered a revolutionary material for the future [4]. Graphene has a unique structure (a hexagonal honeycomb lattice composed of carbon atoms, with s–p hybrid orbitals) and excellent mechanical properties (the theoretical elastic modulus of graphene is as high as 1 TPa, and its fixed tensile strength is 130 GPa), Refs. [5,6,7,8], which can be used as a reinforcing phase to greatly improve the mechanical properties such as strength and stiffness, as well as physical properties such as conductivity and thermal conductivity of metal materials, thereby obtaining high-performance structural and functional materials [9,10,11].

Metal matrix composites are widely used in aerospace and automotive industries for their excellent properties such as high strength-to-weight ratio and low coefficient of thermal expansion. In recent years, fruitful results have been achieved by using nano-phase reinforced metal matrix composites such as carbon nanotubes and graphene [12,13], which have led to the improvement of the composites in terms of hardness, strength and wear resistance [14,15,16,17,18]. Graphene is highly preferred for its excellent mechanical properties such as high Young’s modulus and high strength. Kim et al. [19] confirmed that the load-bearing capacity of graphene is the key to improve the material strength by investigating the strengthening mechanism of single- or two-layer graphene in copper-based (or nickel-based) composites. However, enhancing the strength of metal matrix composites by introducing graphene is often at the expense of their plasticity, and its inverse relationship of strength and toughening limits the further development and application of graphene/metal matrix composites, which is essential. The fundamental reason is that during the deformation of the material, the stress concentration at the interface is prone to crack formation, and the crack expansion cannot be effectively hindered in the subsequent deformation [20]. Numerous studies [21,22] have shown that optimizing the toughness of composites by adjusting the configuration and distribution of graphene is an effective way. Therefore, it is important to elucidate the laws of the influence of the configuration and distribution of graphene in the metal matrix on the mechanical properties of composites for the design of high-strength and high plasticity graphene/metal matrix composites.

It is well known that direct experimental observation of the details and patterns of structural evolution during material deformation is costly and can be limited by modern techniques such as electron and optical microscopy, so new avenues have been explored. Due to the rapid development of computer technology, molecular dynamics (MD) simulations are playing an increasingly important role in the design and development of new materials [23,24,25,26]. MD simulation methods reveal the potential deformation mechanism and the evolution of atomic microstructure in graphene/metal matrix composites, which play a key role in the further design of high-strength and high-toughness composites [27]. Weng et al. [28] investigated the effect of layer thickness on the mechanical properties of graphene/copper matrix composites using MD simulations. Their results showed that graphene can effectively impede the dislocation slip due to its high in-plane strength, and the synergistic effect of graphene/copper interface can significantly enhance the average flow stress of the composites. The synergistic effect of the graphene/Cu interface can significantly enhance the average flow stress of the composite. Shuang et al. [29] found that the continuous transfer of dislocations to graphene leads to local deformation of graphene by the MD simulation method and then proposed three mechanisms of interaction between graphene and dislocations, namely, slip, transmission and reflection. Currently, although preliminary results have been achieved in the study of the mechanical properties of graphene/metal matrix composites, theoretically, the influence laws of the study temperature and the graphene angle on the metal matrix need to be further revealed. In this paper, the mechanical behavior of graphene/aluminum matrix composites is investigated by the MD simulation method. The results of this paper provide some theoretical basis for further design of high performance graphene/aluminum matrix composites.

## 2. Calculation Details

Molecular dynamics is a set of molecular simulation methods that rely on Newtonian mechanics to simulate the motion of a molecular system under certain interaction laws and computers to calculate the phase orbit of a collection of particles to determine the static and dynamic properties of the system, i.e., the thermodynamic and other macroscopic properties of the system. Molecular dynamics simulations were performed using Large-scale Atomic/Molecular Massively Parallel Simulator (LAMMPS) [30]. The Al matrix is crystalline along x, y and z in [100], [010] and [001] [31] directions, respectively. In this paper, the Al–Al interatomic interactions are described by the EAM potential function [32], and the C–C interatomic interactions are described by the AIREBO potential function [33]. The C–Al interatomic interactions are described by the LJ potential function [34]. The potential well depth and zero potential distance parameters between the atoms are shown in Table 1, where they are 0.035 eV as well as 3.014 Å for the Al–C interatomic interactions, respectively [35,36]. It is shown that the cut-off radius (r${}_{cut}$) should be chosen as 3σ or larger, so 9.045 Å is used as the r${}_{cut}$ selection in this paper [37]. The model is subjected to an initial conformational relaxation of 10,000 steps to bring the system atoms to an initial equilibrium state. The time step during the mechanical simulation is set to 0.001 ps with 50,000 iterative steps. The simulation results were visualized and analyzed using Open Visualization Tool (OVITO) software [38]; OVITO is a scientific visualization and data analysis solution for atomistic and other particle-based models. It helps scientists gain meaningful and quick insights from numerical simulation results.

## 3. Results and Discussion

### 3.1. Effect of Temperature on Aluminum Substrate and Graphene Nanosheets

**Figure 1:**
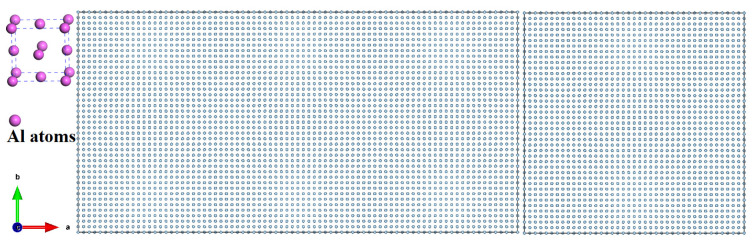
Crystal structure of the model includes 64,000 aluminum atoms; the purple spheres represent aluminum atoms, and the smallest cell composed of purple spheres contains four spheres that occupy the center of the tetrahedral face and the top corner position.

First, we take a perfect aluminum structure as the object of study and systematically investigate its mechanical properties at different temperatures. As shown in Figure 1, we investigate the theoretical tensile stress of aluminum without defects. In this case, a total of 64,000 aluminum atoms are included in the model, and in the figure, we fix one end of it. As shown in the figure, the purple spheres represent aluminum atoms, and the smallest cell composed of purple spheres contains four spheres that occupy the center of the tetrahedral face and the top corner position.

**Figure 2:**
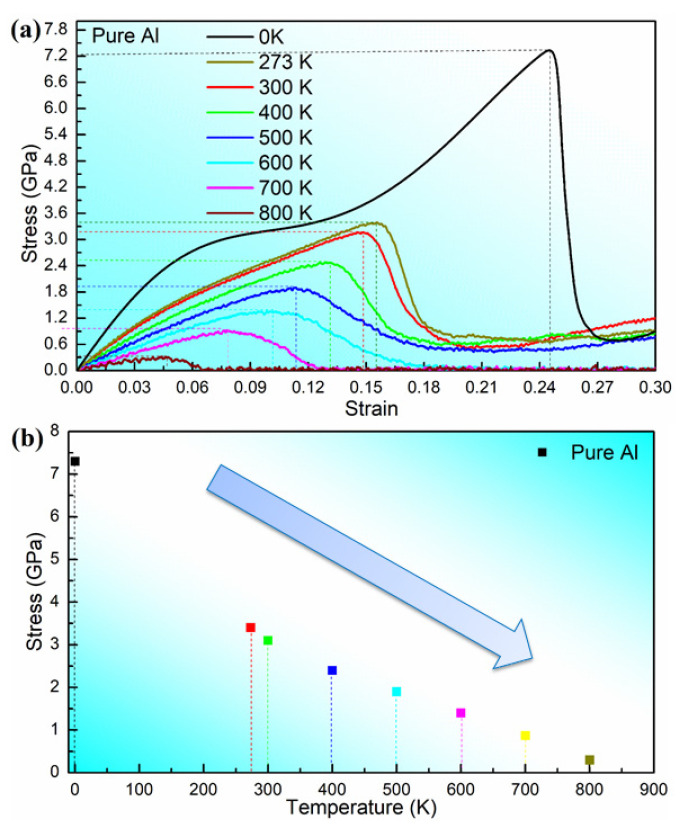
Stress–strain curves (**a**) and values (**b**) of perfect aluminum substrate at different temperatures.

As shown in Figure 2, we simulate the mechanical properties of perfect aluminum crystals at different temperatures. In the figure, we have chosen 0 K, 273 K, 300 K, 400 K, 500 K, 600 K, 700 K and 800 K as the temperature nodes. From the figure, we can see that the tensile properties of perfect aluminum have a decreasing trend with the increase of temperature. The corresponding maximum stresses at 0 K, 273 K, 300 K, 400 K, 500 K, 600 K, 700 K and 800 K are 7.2 GPa, 3.4 GPa, 3.1 GPa, 2.5 GPa, 1.9 GPa, 1.4 GPa, 0.9 GPa and 0.3 GPa, respectively. Experimentally, the mechanical properties of aluminum alloys are affected by temperature [41], and the stress in aluminum alloys decreases with increasing temperature [42,43], which is in agreement with our theoretical results.

**Figure 3:**
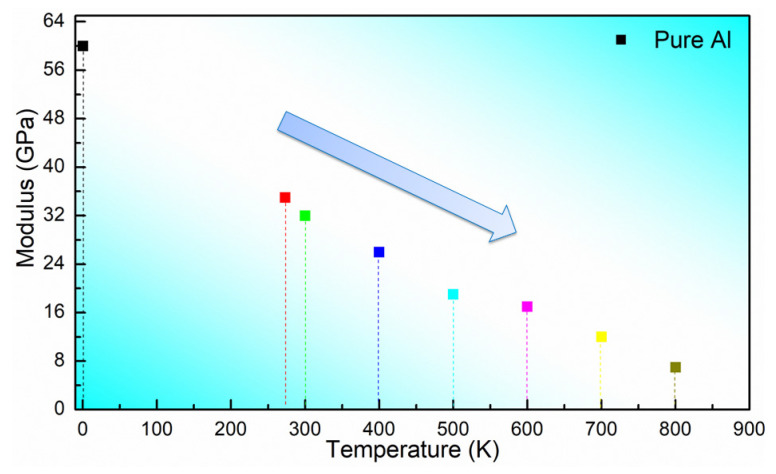
Calculated modulus of perfect aluminum crystals at different temperatures.

As shown in Figure 3 and Table 2, we have calculated the modulus of perfect aluminum crystals at different temperatures. In the figure, we have selected 0 K, 273 K, 300 K, 400 K, 500 K, 600 K, 700 K and 800 K as the temperature nodes to be studied. The calculated results show that the moduli at 0 K, 273 K, 300 K, 400 K, 500 K, 600 K, 700 K and 800 K are 60.1 GPa, 35.2 GPa, 32.4 GPa, 26.1 GPa, 19.3 GPa, 17.2 GPa, 12.1 GPa and 7.2 GPa, respectively. From the figure, we can see that the modulus of the perfect aluminum crystals is around 60 GPa at theoretical case 0 K, which is very close to the theoretical modulus of perfect aluminum crystals (69–71 GPa). We further observe from the figure that the modulus of perfect aluminum decreases approximately linearly with temperature, and the elastic modulus at 800 K decreases by 62.5% relative to room temperature (300 K).

**Figure 4:**
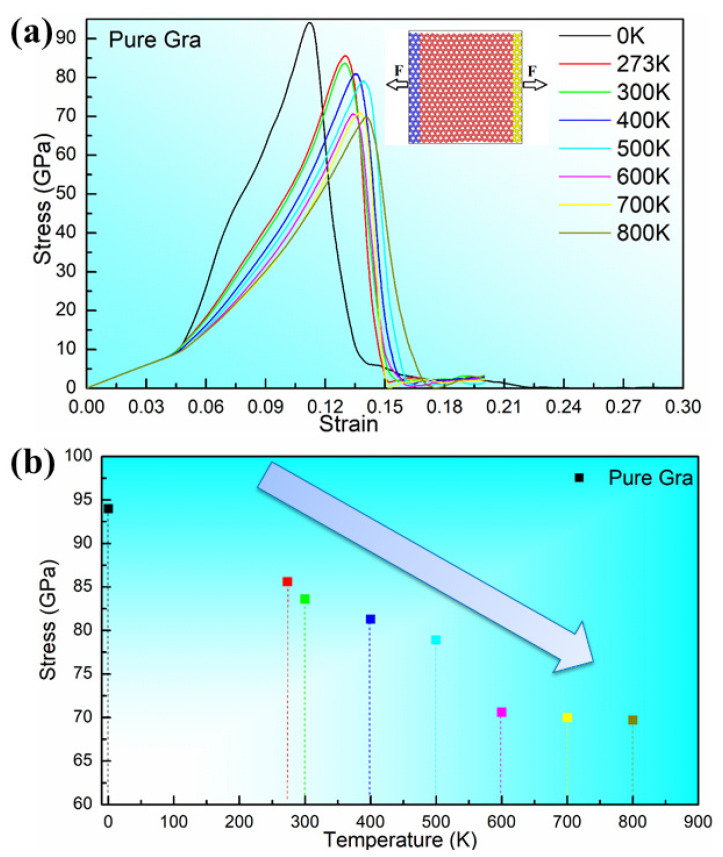
Stress–strain curves (**a**) and values (**b**) of perfect graphene at different temperatures.

Before studying the composite properties, we investigate the mechanical behavior of perfect graphene. As shown in Figure 4 and Table 3, the theoretical tensile stresses of perfect graphene at different temperatures were studied. In this case, a total of 51,200 C atoms are included in the model, and in the figure, we fix one end of it. As shown in the figure, the blue area spheres as well as the yellow area spheres are the atoms at the fixed end as well as the atoms at the mobile end, respectively. As shown in Figure 4, we investigated the stress–strain relationships at 0 K, 273 K, 300 K, 400 K, 500 K, 600 K, 700 K and 800 K, respectively. We can see from Figure 4 that the highest point of the stress–strain curve tends to decrease as the temperature increases, except for the overall rightward shift of the curve as the temperature increases.

**Figure 5:**
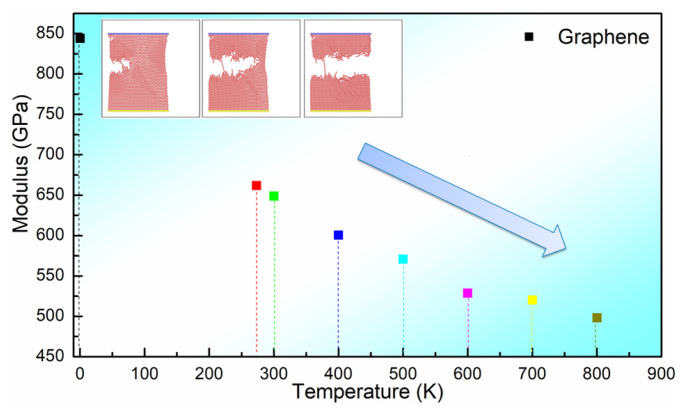
Calculated modulus of perfect graphene at different temperatures.

As shown in Figure 5, we have calculated the modulus of perfect graphene at different temperatures. In the figure, we have selected 0 K, 273 K, 300 K, 400 K, 500 K, 600 K, 700 K and 800 K as the temperature nodes. From the figure, we can see that the modulus of perfect graphene at the theoretical case 0 K is around 850 GPa, which is very close to the theoretical modulus of perfect graphene (1 TPa = 1000 GPa) [44]. We further observe from the figure that the modulus of perfect graphene decreases approximately linearly with temperature, and the elastic modulus decreases by 23.2% at 800 K relative to room temperature (300 K).

### 3.2. Effect of Graphene Distribution on the Mechanical Properties of Graphene/Aluminum Composites

**Figure 6:**
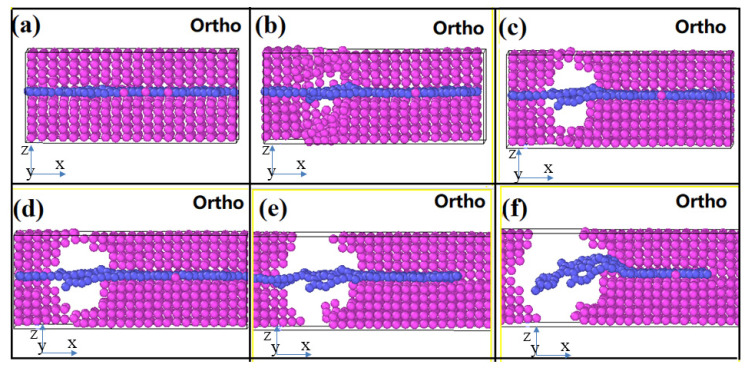
Diagram of single-layer graphene/aluminum composite in the tensile failure process, (**a**–**f**) represent the process of matrix cracking until material failure during stretching.

After discussing the mechanical properties of perfect graphene and the aluminum matrix, we investigate the mechanical properties and failure behavior of perfect graphene/aluminum composites using molecular dynamics. In our previous work [45], we found that Al (111) can be better compounded with graphene (001). We sectioned Al (111) as well as graphene (001) with lattice constants of 2.86 Å and 2.46 Å, respectively, and expanded their protoplasts by a factor of 5 (14.30 Å) and 6 (14.76 Å), respectively. As shown in Figure 6, we choose a small cell model of the graphene/Al composite, as well as its supercell model, in which the lattice fitness ratio of aluminum to graphene is kept around 3.22%. In the figure, we choose a single layer of graphene as the reinforcement of the aluminum matrix, and it can be seen from the figure that as the strain increases, the simulated tensile process does not break the graphene, but rather the graphene and the aluminum matrix shear, and then the graphene “pulls out” from the aluminum matrix.

**Figure 7:**
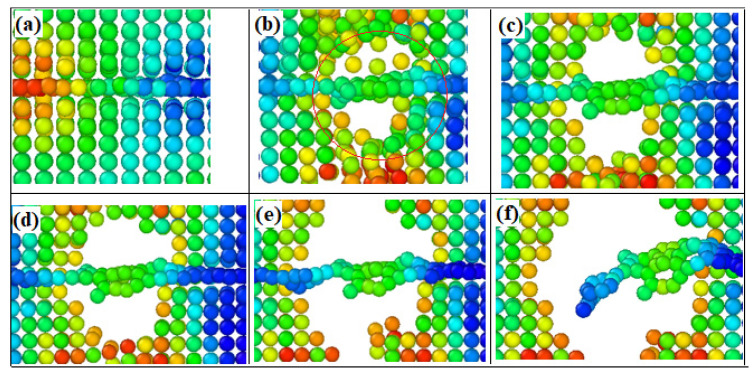
Stress distribution of single-layer graphene/aluminum composites during tensile failure, (**a**–**f**) represent the process of matrix cracking until material failure during stretching.

From Figure 6 and Figure 7, we can see that the hole defects first appear at the graphene–substrate interface during the simulated stretching process. With the increase of strain, the holes expand and then cracks are generated. As the crack expands, the matrix cracks, but the composite material has not yet failed, and the matrix is connected by graphene as a “bridge”. As the strain increases further, the graphene is withdrawn from the side of the matrix and the material fails. In Figure 7, the shades of color represent the stratification of the stress, with the red area representing the maximum stress and the blue area representing the minimum stress. From Figure 7, we can see that at the beginning of the strain, the stress is mainly concentrated at the interface between the graphene and the matrix, and as the matrix cracks, the stress is finally concentrated on the graphene as the “bridge”.

**Figure 8:**
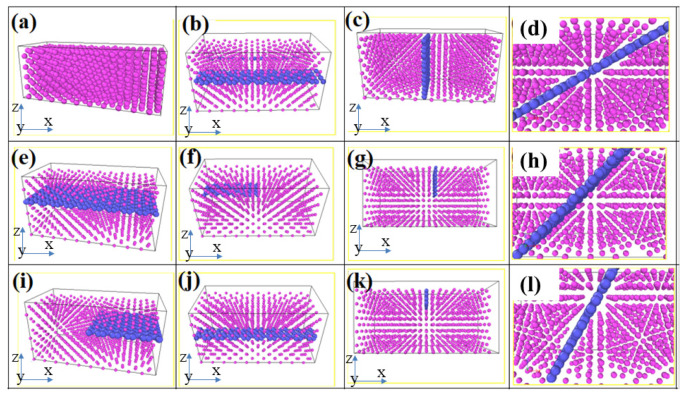
Schematic representation of graphene distribution in graphene/aluminum composites.

**Figure 9:**
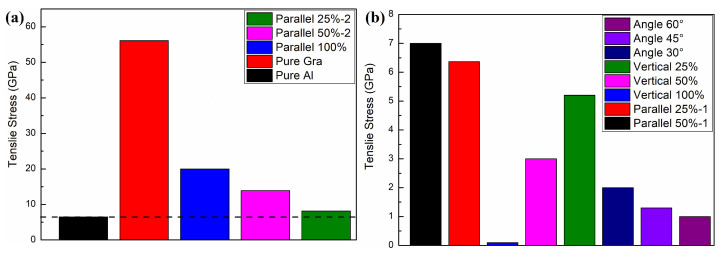
Mechanical properties of different forms of graphene/aluminum composites. The tensile stress of structures with different area ratios of graphene parallel to the stretching direction are shown in (**a**), and the tensile stress of structures with different graphene angles to the stretching direction are shown in (**b**).

As shown in Figure 8, we further investigated the effect of graphene size as well as angle on the mechanical properties of graphene/aluminum composites. Figure 8a shows a perfect aluminum matrix; Figure 8e shows a composite model with 100% graphene-to-matrix area ratio; Figure 8b,i show a composite model with 50% graphene-to-matrix area ratio in the x-direction and y-direction, respectively; Figure 8f,j show a composite model with 25% graphene-to-matrix area ratio in the x-direction and y-direction, respectively. Figure 8c,g,k show models of composites with graphene-to-matrix area ratios of 100%, 50% and 25% in the vertical and tensile directions, respectively; Figure 8d,h,l show models of composites with graphene at angles of 30${}^{\circ }$, 45${}^{\circ }$ and 60${}^{\circ }$ with the tensile direction, respectively. As shown in Figure 9, we calculate the mechanical properties of the above models separately. From Figure 9, we can see that in the parallel x-direction, the tensile stress tends to increase as the percentage of graphene area increases; in the vertical x-direction, the tensile stress tends to decrease as the percentage of graphene area increases; in the angle with the x-axis, the tensile stress tends to decrease as the angle between graphene and the x-axis increases. At the same time, we can also see from the figure that above the dashed line, the composite tensile stresses are all greater than the single crystal aluminum tensile stresses, which have a strengthening effect.

### 3.3. Multilayer Graphene in Graphene/Aluminum Composite Failure Behavior

**Figure 10:**
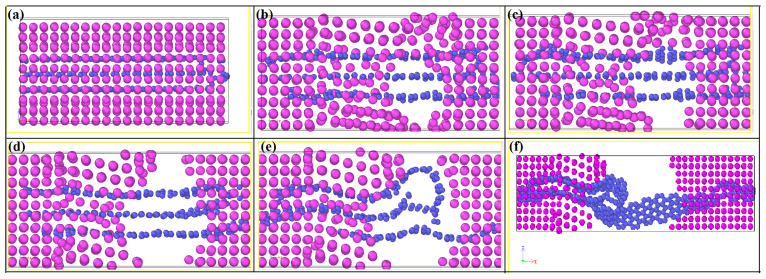
Schematic diagram of the tensile failure process of the three-layer graphene/aluminum composite. (**a**–**f**) represent the process of matrix cracking until material failure during stretching.

As shown in Figure 10, we investigate the tensile fracture behavior of three-layer graphene in graphene/aluminum composites. From Figure 10, we can see that at the beginning of strain loading, “hole defects” appear first at the contact between the outer layer of graphene and the matrix, and as the strain continues to increase, the “hole defects” show a tendency to expand gradually, and finally the matrix fractures. At this time, the three layers of graphene connected the two sides of the matrix, acting as a “bridge”, so that the composite material did not fail. With the further increase of strain, the bottom graphene layer slowly withdraws from the matrix, and at this time, the strength of the composite comes from the shearing action between the inner layer graphene and outer layer graphene and the shearing action between graphene and the surface of the matrix. With further increase in strain, the underlying graphene is withdrawn from the matrix as well as from the intermediate graphene, leading to complete failure of the composite. The strong toughening and fracture behavior of multilayer graphene/Al composites were investigated experimentally by Wu et al. [46,47], and their results indicate that the interlayer bonding force is weaker than the graphene–matrix interface bonding force, resulting in the mislayer slip of graphene, which is in agreement with our calculated results.

**Figure 11:**
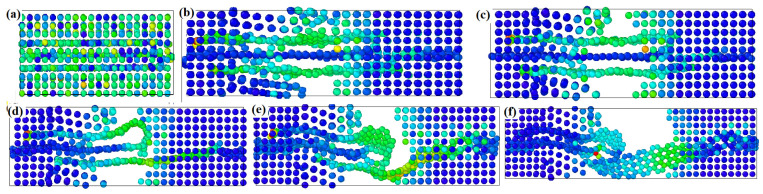
Stress distribution of three-layer graphene/aluminum composite during tensile failure. (**a**–**f**) represent the process of matrix cracking until material failure during stretching.

From Figure 11a,b, we can see that at the beginning of the stretching, there is a yellow-green distribution in the aluminum substrate as well as in the graphene, which means that the stress is distributed in the aluminum substrate as well as in the graphene at this time. As the strain increases, we can see from Figure 11d–f that the yellow-green color is mainly distributed between the graphene layers and between the outer graphene and the aluminum substrate. From Figure 11, we can see that at the beginning of strain loading, the stresses are mainly concentrated on the two outer graphene layers. With further increase of strain, the stresses are concentrated between the outer graphene layers and the matrix. It is further observed that the bottom graphene layer bears the main stress before the failure of the composite. At the moment of composite failure, the stresses are mainly concentrated at the edge of the graphene and the matrix. The study of the failure behavior of multilayer graphene in graphene/aluminum composites provides some theoretical references for further research on graphene-reinforced metal matrix composites, thus enhancing the mechanical properties.

## 4. Conclusions

The effects of temperature and graphene on the mechanical properties of an aluminum matrix were investigated by molecular dynamics methods. The stress in the aluminum matrix decreased with increasing temperature, and a similar trend was observed for the modulus. In the study of monolayer graphene-reinforced aluminum substrates, it was found that holes first appeared at the graphene–substrate interface. As the strain increases, the holes expand, and further expansion of the crack leads to cracking of the substrate, with graphene as the “bridge” connection and stress concentrated on the “bridge” of graphene. The study of the three-layer graphene-reinforced aluminum substrate found that the shear between the outer graphene layer and the substrate occurs late in the strain, which is the main factor for stress retention in the material. In studying the effect of graphene size and angle on the mechanical behavior of graphene/Al composites, we found that in the parallel x-direction, the tensile stress tends to increase with increasing percentage of graphene area. In the perpendicular x-direction, the tensile stress tends to decrease with the increase of the percentage of graphene area. In the angle with the x-axis, the tensile stress tends to decrease with increasing the angle of graphene with the x-axis. By investigating the effects of temperature and graphene on the mechanical properties of an aluminum matrix, it provides some theoretical references for the study of graphene-reinforced metal matrix composites experimentally.

## Figures and Tables

**Table 1:** Lennard–Jones (L–J) potential parameter for atomic interactions.

	Interacting	Atoms	Types
L–J potential function	Al–Al [39]	C–C [40]	Al–C [35,36]
σ/Å	2.620	3.407	3.014
η/eV	0.416	0.003	0.035

**Table 2:** Modulus and stress values of perfect aluminum crystals at different temperatures.

	0 K	273 K	300 K	400 K	500 K	600 K	700 K	800 K
Modulus (GPa)	60.1	35.2	32.4	26.1	19.3	17.2	12.1	7.2
Stress (GPa)	7.2	3.4	3.1	2.5	1.9	1.4	0.9	0.3

**Table 3:** Modulus and stress values of perfect graphene at different temperatures.

	0 K	273 K	300 K	400 K	500 K	600 K	700 K	800 K
Modulus (GPa)	844.1	661.8	648.9	600.7	570.8	528.9	520.3	498.2
Stress (GPa)	94.1	85.6	83.7	80.9	79.2	70.8	68.2	67.3

## Data Availability

The data that supports the findings of this study are available within the article.
